# Quantitative dynamic contrast-enhanced magnetic resonance imaging in a VX2 rabbit liver tumour model using different gadolinium-based contrast agents: comparison of DCE-MRI quantitative results between Magnevist and Eovist

**DOI:** 10.1259/bjrcr.20160099

**Published:** 2017-04-12

**Authors:** Yule Zhang, Hongsheng Liu, Weiqiang Xiao, Liling Zhu, Na Wang, Xuehua He, Zhimin Jiang, Buyun Guan

**Affiliations:** ^1^Ultrasound, Guangzhou Women and Children's Medical Center, Guangzhou Medical University, Guangzhou, China; ^2^MR, Guangzhou Women and Children's Medical Center, Guangzhou Medical University, Guangzhou, China; ^3^Mkt, GE Healthcare, Guangzhou, China

## Abstract

Quantitative dynamic contrast enhanced MRI (DCE-MRI) can offer information related to tumour perfusion and permeability (K^trans^), rate constant (K_ep_), extravascular extracellular volume fraction (V_e_) and distribution volume (V_d_). Different types of gadolinium-based contrast agents (GBCAs) may traverse the vascular wall with different velocities owing to their physicochemical characteristics. The purpose of this article was to compare the DCE-MRI quantitative results (K^trans^, K_ep_, V_e_ and V_d_) between Magnevist and Eovist in a VX2 rabbit liver tumour model. Sixteen rabbits (body weight, 3 Kg; random gender) containing implanted hepatic VX2 carcinomas were randomly divided into two groups based on the regimen of MRI contrast agent administered, eight rabbits in each group. All rabbits underwent a liver DCE-MRscan before tumour transplantation. Fourteen days after tumour transplantation, the eight rabbits in Group A (Magnevist group) underwent a liver DCE-MR scan in a 3.0 T Magnetom Verio MR scanner (Siemens Healthcare, AD, Germany) after the administration of Magnevist at the flow rate of 1 ml s^–1^. The Group B rabbits underwent the same scan except for the administration of Eovist at the same flow rate. Twenty-four hours after the initial DCE-MRI, repeat DCE-MRI was performed with the cross-over GBCA at the same flow rate in each group. Every rabbit received 0.6 ml GBCA (0.2 ml Kg^–1^) during each DCE-MRI. K^trans^, K_ep_, V_e_ and V_d_ were measured in the tumour lesion and compared with normal liver tissue in the same slice. A pathologic examination was also performed. Hepatocellular carcinoma was diagnosed in all 16 rabbits by pathologic examination. There were no significant differences in K^trans^, V_e_, K_ep_ and V_d_ between the two groups of rabbits (*p* > 0.05). The K^trans^, V_e_, K_ep_ and V_d_ of the VX2 rabbit liver tumour model were significantly higher than the normal liver parenchyma (0.742 ± 0.086 *vs* 0.027 ± 0.002, 7.345 ± 0.043 *vs* 6.721 ± 0.035, 0.101 ± 0.005 *vs* 0.101 ± 0.005, 0.419 ± 0.083 *vs* 0.037 ± 0.005, respectively; *p* < 0.01). The K^trans^, V_e_ and V_d_ of Eovist group were significantly higher compared with the values in the Magnevist group (0.116 ± 0.016 *vs* 0.010 ± 0.002, respectively, *p* < 0.01; 0.101 ± 0.005 *vs* 0.004 ± 0.0009, respectively, *p* < 0.01; 0.419 ± 0.083 *vs* 0.037 ± 0.005, respectively, *p* < 0.001). There was no significant difference in K_ep_ between the Eovist and Magnevist groups (7.345 ± 0.043 *vs* 6.721 ± 0.035, respectively; *p* > 0.05). In the VX2 rabbit liver tumour model, DCE-MRI performed with different types of GBCA can develop different quantitative results with respect to K^trans^, V_e_ and V_d_. The liver-specific GBCA, Eovist, is more sensitive than the general GBCA, Magnevist, in detecting tumour perfusion and permeability.

DCE-MRI can make quantitative assessment of tissue vessel density, integrity and permeability without invasion.^1^ We could apply the data to the study of hypoxia, angiogenesis and biomarker evaluation. DCE-MRI has been successfully applied in the studies of animal and human tumour models, monitoring of treatment response, characterization of predictive prognostic factors and evaluation of the efficacy of novel treatment developments in the areas of central nervous system, liver and prostate tumours. There has been more recent attention paid to hepatic disease research with DCE-MRI, but its application to the diagnosis of hepatocellular carcinoma (HCC) after surgery is still in the basic research stage.

DCE-MRI relies on fast repeated image acquisition before, during and after the rapid intravenous administration of a low molecular weight, gadolinium-based contrast medium. The gadolinium-based contrast agents (GBCAs) used for DCE-MRI are paramagnetic agents. The two most commonly used GBCAs in liver DCE-MRI are gadopentetate dimeglumine [Gd-diethylenetriamine pentaacetic acid (DTPA)] and gadoxetic acid disodium (Gd-EOB-DTPA). After intravenous bolus injection, each GBCA quickly spreads to the extracellular space where it can be absorbed into the blood and filtered by glomerular filtration.^2^ In general, the more abundant the blood supply, the higher the vascular permeability, which leads to an enhance signal on *T*_1_ weighted MRI.

Gd-EOB-DTPA, a derivative of Gd-DTPA, is a liver-specific contrast agent used in the detection of liver tumours, which can spread to the extracellular space via the blood and is filtered by glomerular filtration (similar to Gd-DTPA). Gd-EOB-DTPA can enter the hepatocyte approximately 2 min after injection, and is then secreted into the bile and eliminated by the bile ducts. These two excretion pathways each account for approximately 50% of the excretion of Gd-EOB-DTPA.^3^ After absorption by the hepatocytes, the Gd-EOB-DTPA can shorten the *T*_1_ relaxation time, resulting in an increase in signal on *T*_1_ weighted MRI in cases of normal liver parenchyma and benign liver tumours.^4^ However, since there is no special absorption of contrast media in malignant liver tumours or non-hepatocyte tumours (such as metastases), a decrease in signal will occur on *T*_1_ weighted images.^5^ Therefore, performing DCE-MRI with Gd-EOB-DTPA can improve the detection rate of liver tumours.

The purpose of this study was to compare quantitative DCE-MRI results using K^trans^, K_ep_, V_e_ and V_d_ between Magnevist and Eovist in a VX2 rabbit liver tumour model.

## Materials and Methods

### Animal preparation and treatment

#### Grouping

Our protocol was approved by our Institutional Review Board. Seventeen New Zealand rabbits (average body weight, 3 Kg; random gender) were provided by the GuangDong Medical Laboratory Animal Center. Sixteen of the 17 rabbits were randomly divided into two groups of eight [Group A (the Magnevist Group) and Group B (the Eovist Group)] based on the regimen of GBCA used. All animals were housed in an approved, climate-controlled, animal care facility and were given standard rabbit chow and water *ad libitum*.

#### Inoculation with tumour cells

The additional rabbit (the 17th rabbit), who had improved tumour-burdened VX2 and VX2 cell lines, was used for this phase of the experiment. The tumour cells were inoculated into the subcutaneous tissue of the rabbit thigh to promote tumour formation and generation. Approximately 2 weeks later, the tumour was removed in 1–2 mm sized pieces which were then placed on a plate using an 18G needle.

The 16 rabbits were narcotized with 3% pentobarbital sodium (1 ml kg^–1^) by intravenous anesthesia and positioned on the CT scanning bed in the supine position. Under CT guidance, each rabbit liver was injected with approximately two or three blocks of the prepared tumour strains to a Pinpoint Pierce depth of approximately 1.5~2.0 cm. To ensure a successful rate of implantation, the right and left lobes of each rabbit liver were implanted with tumour foci and the needle passage was sealed with gelatin sponge. A CT scan was then performed to exclude any active bleeding. After each rabbit recovered, the animal was sent back to its holding pen to continue breeding.

#### Treatment

The 16 rabbits underwent non-enhanced and DCE-MRI scans before inoculation with the VX2 cell line. After inoculation, the rabbits in Group A underwent DCE-MRI after the injection of Gd-DTPA. Twenty-four hours later, they were scanned again after injection of Gd-EOB-DTPA. In contrast, Group B rabbits underwent DCE-MRI after the injection of Gd-EOB-DTPA and 24  h later underwent repeat DCE-MRI with Gd-DTPA. The two groups of animals were scanned using identical pulse sequences and scan parameters.

Fourteen days after inoculation, the rabbits underwent weekly unenhanced MRI scans to observe tumour size. When the tumour reached >1.0 cm in diameter, that rabbit immediately underwent both unenhanced and DCE-MRI scans. Group A rabbits were scanned after the injection of Gd-DTPA for the first scan. Twenty-four hours later, they were again scanned after the injection of Gd-EOB-DTPA. Group B rabbits, in contrast to Group A rabbits, were first scanned with Gd-EOB-DTPA and then scanned again with Gd-DTPA. The two groups of animals were scanned identically using the same pulse sequences and scan parameters. After MRI examination, the 16 rabbits were euthanized via the air embolism method. Pathological and MRI results underwent comprehensive quantitative analysis.

### DCE-MRI scanning methods and parameters used

All DCE-MRI scans were performed using a 3.0 T Magnetom Verio MR scanner (Symphony model, Siemens Healthcare, AD, Germany). Scanning parameters were as follows: FLASH *T*_1_ weighted image: TR/TE = 210 ms/2.6 ms; slice thickness, 5.5 mm; gap, 0.6 mm; slices, 10–16; vision (FOV), 28 cm ×21 cm; matrix size, 512 × 384; NEX, 4; acquisition time, 40 s; TSE *T*_2_ weighted image sequence: TR/TE = 4000 ms/101 ms; slice thickness, 5.5 mm; gap, 0.6 mm; number of slices, 10–16; FOV, 28 cm × 21 cm; matrix size, 512 × 384; NEX, 4; acquisition time, 40 s.

MR perfusion imaging used the Vibe sequence, axial scanning, TR/TE = 2 ms/5 ms; slice thickness, 5.0 mm; gap, 0.2 mm; phase, 60; number of slices in each phase, 10; FOV, 16 cm × 13.5 cm; matrix size, 160 × 135; NEX, 1; flip angle, 12; acquisition time, 6 s.

Dynamic contrast-enhanced scans were performed using a high-pressure syringe for injection of GBCA. The contrast agent was rapidly injected via the rabbit’s ear vein. The Group A rabbits were first injected with Gd-DTPA (Magnevist, Bayer Schering; Gadopentetate Dimeglumine, Gd-DTPA) and, after 24 h, they were injected with Gd-EOB-DTPA (Eovist, Bayer Schering; Gadoxetic acid, Gd-EOB-DTPA), at a dose of 0.2 mmol kg^–1^ and an injection rate of 1 ml s^–1^. Scanning was begun simultaneously with the injection of contrast agent, followed by a 5 ml saline flush to ensure full use of the contrast agent. Group B rabbits were injected in the opposite order. After DCE-MRI, a regular enhanced scan was performed with axial and coronal FLASH *T*_1_ weighted image sequences by the same parameters with modified non-contrast *T*_1_ weighted imaging.

#### Scan parameter measurements

After selecting the maximum level of the lesion, the maximum slope of increase variation and time-signal intensity curve were used to analyse the aorta, spleen, normal liver parenchyma, tumour and necrotic tissue peripheral enhancement area. The capacity transfer constant (K^trans^, unit min^–1^) and the rate constant (K_ep_, unit min^–1^) were also calculated. When performing the measurements, the radiologist tried to avoid blood vessels, bile ducts and artifacts, while measuring at the maximum level of lesion diameter. Each region of interest area was chosen so as to be > 20 ± 5 mm^2^ and to cover more than 50 pixels. The measurement of non-inoculated liver tissue was performed in the same region of interest.

### Gross pathologic specimen analysis

After completion of the experiment, the rabbits were euthanized and the entire liver was removed from each rabbit. Each specimen was visually inspected for the presence of tumour tissue, shape and colour as well as evaluated for the presence or absence of peritumoral nodule size, shape and number. Based on the MRI images, the liver was sectioned in the direction of the scan level to provide the maximum amount of tumour per section. The specimens were then immersed in 10% formalin, fixed, paraffin embedded, cut into 4 um sections and stained with conventional hematoxylin and eosin (H&E) staining.

After H&E staining, each specimen was observed under optical light microscope for the following content: ① tumour growth: tumour with or without capsule formation or capsular invasion, growth characteristics of the lesion edge, the presence or absence of a tumour nodule, presence or absence of branch portal vein tumour thrombosis, presence or absence of bile duct invasion and intrahepatic metastasis; ② signs of tumour angiogenesis (i.e., lack of structural integrity and intercellular connections, weak wall, lack of arrangement of endothelial cells in layers, lack of smooth muscle, incomplete basement membrane permeability) and ③ bleeding, fibrosis, calcification and intratumoral sinus dilation.

### Statistical analysis

SPSS 14.0 statistical software was used for all statistical analysis of the perfusion imaging quantitative results and the results were expressed as mean ± standard deviation. Perfusion imaging quantitative results were based on the SPSSR scan of the two groups of animals and indicators that were compared. These indicators were compared using paired t-test and included type of GBCA used (i.e., Magnevist *vs* Eovist), variation between normal tissue *vs* liver lesion maximum slope of increase, the time-signal intensity curve, the capacity transfer constant (K^trans^, unit min^–1^) and the rate constant (K_ep_, unit min^–1^). A control group of normal rabbit liver DCE-MRI scans was used as a metric for comparison with the experimental group, using a paired t-test. A *p*-value <0.05 was considered statistically significant.

## Results

Hepatocellular carcinoma was diagnosed in all 16 rabbits by pathologic examination. Two of the 16 rabbits (12.5%) had metastasis and one (6.3%) had hemorrhage. There were no significant differences in K^trans^, V_e_, K_ep_ or V_d_ between Group A and Group B (*p* > 0.05).

**Figure 1.**
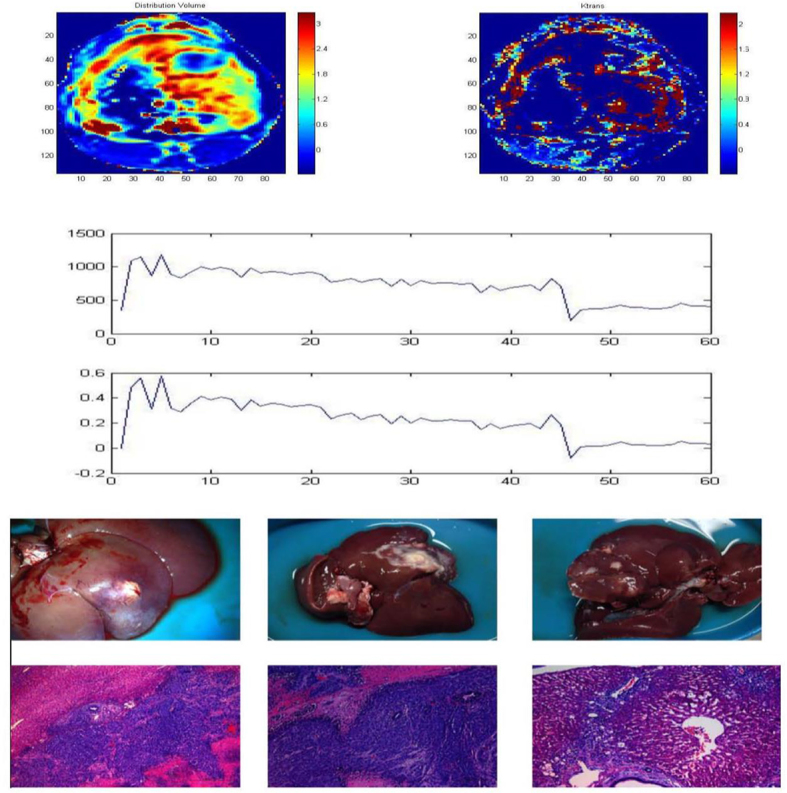
DCE-MRI with Eovist.

**Figure 2.**
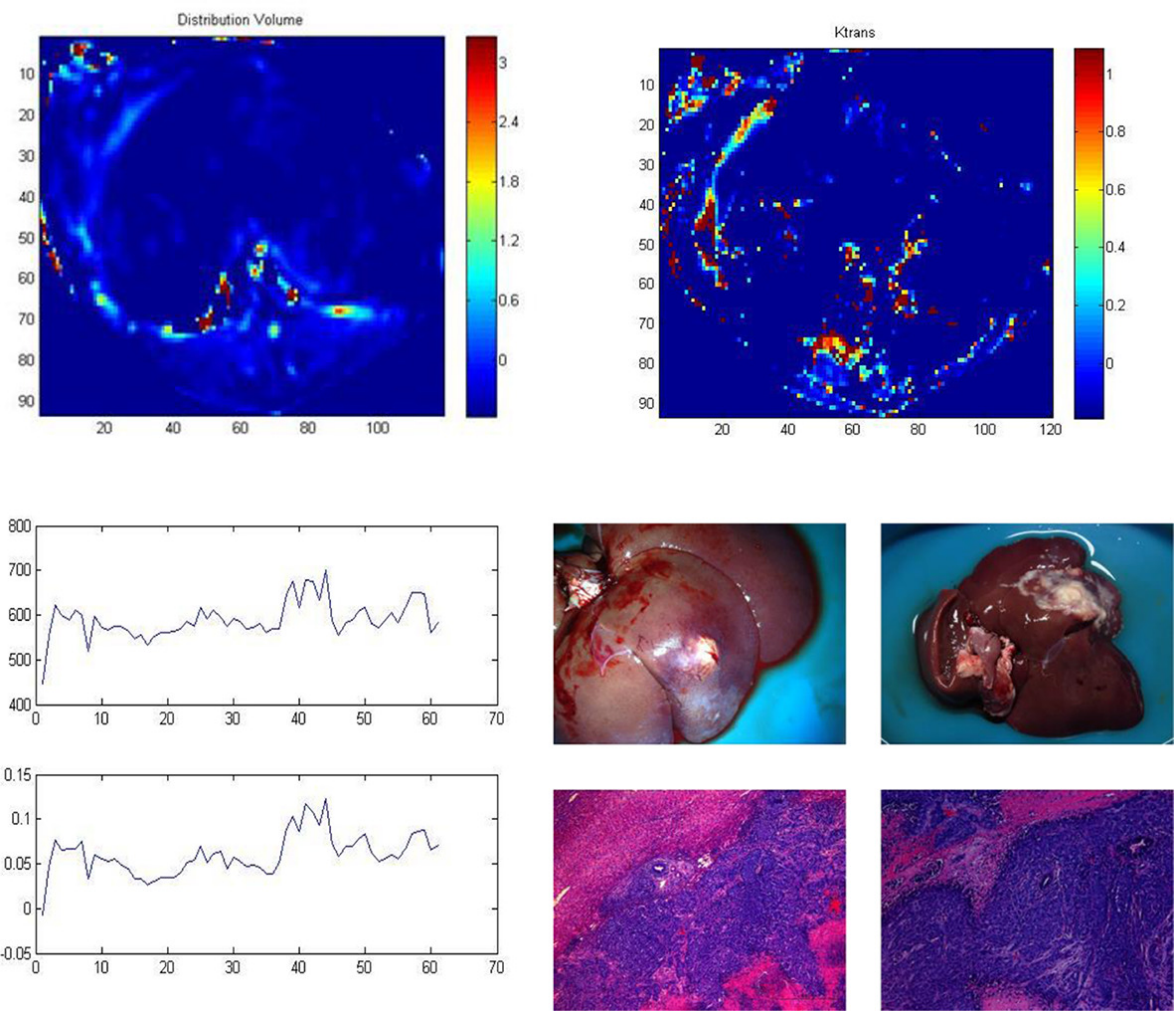
DCE-MRI with Magnevist.

The K^trans^, V_e_, K_ep_ and V_d_ of the VX2 rabbit liver tumour model were significantly higher compared with normal liver parenchyma (0.742  ± 0.086 *vs* 0.027 ± 0.002, 7.345 ± 0.043 *vs* 6.721 ± 0.035, 0.101 ± 0.005 *vs* 0.101  ±  0.005, 0.419  ±  0.083 *vs* 0.037 ± 0.005, respectively; *p* < 0.01; Tables 1,2; Figures 1,2).

**Table 1.** DCE-MRI with Magnevist: comparison between normal liver parenchyma and liver tumour

	Normal	Tumour	*p*-value
K^trans^	0.003 ± 0.0016	0.027 ± 0.002	<0.01
K_ep_	1.125 ± 0.017	6.721 ± 0.035	<0.01
V_e_	0.0027 ± 0.008	0.004 ± 0.0009	<0.01
V_d_	0.029 ± 0.007	0.037 ± 0.005	<0.01

**Table 2.** DCE-MRI with Eovist: comparison between normal liver parenchyma and liver tumours

	Normal	Tumour	*p*-value
K^trans^	0.003 ± 0.0012	0.742 ± 0.086	<0.01
K_ep_	1.253 ± 0.021	7.345 ± 0.043	<0.01
V_e_	0.0027 ± 0.006	0.101 ± 0.005	<0.01
V_d_	0.125 ± 0.012	0.419 ± 0.083	<0.01

The K^trans^, V_e_ and V_d_ of the Eovist group were significantly higher compared with the parameters in the Magnevist group (0.742 ± 0.086 *vs* 0.027 ± 0.002, *p *< 0.01; 0.101 ± 0.005 *vs* 0.004 ± 0.0009, *p *< 0.01; 0.419 ± 0.083 *vs* 0.037 ± 0.005, *p* < 0.01, respectively). There was no significant difference in K_ep_ between the Eovist group and the Magnevist group (7.345 ± 0.043 *vs* 6.721 ± 0.035, *p* > 0.05; Table 3).

**Table 3.** DCE-MRI: comparison between Magnevist and Eovist in liver tumours

	Eovist	Magnevist	*p* value
K^trans^	0.742 ± 0.086	0.027 ± 0.002	<0.01
K_ep_	7.345 ± 0.043	6.721 ± 0.035	>0.05
V_e_	0.101 ± 0.005	0.004 ± 0.0009	<0.01
V_d_	0.419 ± 0.083	0.037 ± 0.005	<0.01

## Discussion

Our results showed the feasibility of DCE-MRI in liver tumour diagnosis. The DCE-MRI performed with the liver-specific GBCA, Gd-EOB-DTPA, provided more accurate information that the general GBCA owing to its liver specificity, as it is absorbed by the hepatocytes and secreted into the bile ducts. Randomized, controlled cross-over injection of contrast media in the two groups of rabbits was designed to minimize test result deviation caused by drug residues. In order to minimize the drug residues from the first dose, we used an injection interval of 24 h since the elimination half-life of Magnevist is approximately 70 min and that of Eovist is approximately 55 min using standard dosing.^6,7^ In general, we found no significant difference in DCE-MRI parameters between the two groups of animals (Tables 4 and 5).

**Table 4.** DCE-MRI with Magnevist: comparison between Groups A and B

	Group A	Group B	*p*-value
K^trans^ (normal)	0.003 ± 0.0014	0.003 ± 0.0017	>0.05
K_ep_ (normal)	1.129 ± 0.018	1.120 ± 0.015	>0.05
V_e_ (normal)	0.0028 ± 0.008	0.0029 ± 0.008	>0.05
V_d_ (normal)	0.031 ± 0.008	0.027 ± 0.005	>0.05
K^trans^ (tumour)	0.028 ± 0.002	0.026 ± 0.002	>0.05
K_ep_ (tumour)	6.735 ± 0.035	6.718 ± 0.035	>0.05
V_e_ (tumour)	0.004 ± 0.0009	0.004 ± 0.0009	>0.05
V_d_ (tumour)	0.039 ± 0.005	0.035 ± 0.005	>0.05

**Table 5.** DCE-MRI with Eovist: comparison between Groups A and B

	Group A	Group B	*p*-value
K^trans^ (normal)	0.003 ± 0.0013	0.003 ± 0.0011	>0.05
K_ep_ (normal)	1.232 ± 0.022	1.265 ± 0.022	>0.05
V_e_ (normal)	0.0027 ± 0.007	0.0027 ± 0.006	>0.05
V_d_ (normal)	0.134 ± 0.013	0.122 ± 0.011	>0.05
K^trans^ (tumour)	0.763 ± 0.089	0.731 ± 0.083	>0.05
K_ep_ (tumour)	7.356 ± 0.043	7.341 ± 0.043	>0.05
V_e_ (tumour)	0.106 ± 0.005	0.100 ± 0.005	>0.05
V_d_ (tumour)	0.421 ± 0.083	0.415 ± 0.083	>0.05

DCE-MRI quantitative parameters were derived from gadolinium concentration *vs* time curves. They were fitted to a two-compartment pharmacokinetic model as proposed by Tofts et al.^8^ This quantitative analysis involved evaluation of several combinations of principal kinetic parameters including the transfer constant (K^trans^), the extravascular extracellular space (EES), the fractional volume (v_e_), the rate constant (k_ep_), and the fractional plasma volume (v_p_). The K^trans^ (min^−1^) represents the transendothelial transport of contrast medium from the vascular compartment to the tumour interstitium. The k_ep_ (min^−1^) reflects the reverse transport of contrast medium back to the vascular space. K^trans^ and v_e_ relate to the tissue’s basic physiology, whereas, the rate constant (k_ep_) is the ratio of the transfer constant to the EES.^8^

k_ep_ = K^trans^/V_e_. 

The liver receives a dual blood supply from the hepatic artery and the portal veins. The contrast agent enters the extracellular space via rapid blood flow due to the high permeability of the liver capillaries, and quickly exchanges between intravascular and extravascular spaces with the result that the blood volume entering the liver and extracellular volume are considered as one space.^9^ Therefore, compared with the general contrast agent, Gd-DTPA, data measured by dual-input single compartment model is well matched with the liver parenchyma. For malignant liver tumours, however, the reduction in capillary permeability surface area and the slow bidirectional endovascular exchange require separate measurements. Thus, the plasma and EES can be regarded as two independent spaces which comprise the dual-input two-compartment model. Using the liver-specific contrast agent, Gd-EOB-DTPA, in normal liver parenchyma, the intracellular space is needed to comprise another dual-input, two-compartment model, which includes both plasma volume and intra-extracellular space volume.^10^ When using a liver-specific contrast agent to evaluate liver tumours, a dual-input three-compartment model is generated as the plasma, intracellular space and extracellular space are considered as three separated spaces. This model can be applied in any case, but in this experiment, we used a dual-input, two-compartment model as it is the most popular model currently in use.

Primary HCC, the most common primary hepatic tumor, is also the third most common cause of cancer death worldwide.^11^ The liver is the largest solid organ in the body and has a dual blood supply. It receives 75% of its blood supply from the portal veins, which are the primary blood supply in benign nodular lesions. The remaining 25% of the liver’s blood supply is from the hepatic artery, which is the primary blood supply of HCC and is indicative of its malignant nature. As HCC grows, according to Tajima et al.,^12^ the blood supply of normal hepatic artery and portal veins decrease gradually, while abnormal proliferation of the hepatic artery gradually increases. While conducting a comparison study of DCE-MRI parameters between micro-vessel density, vascular endothelial growth factor, and P53 protein, Wang et al.^13^ found that DCE-MRI can reflect the number and activity of angiogenesis in HCC. In addition, Hsu et al.^14^ showed a correlation between K^trans^ value and treatment response/survival in HCC patients. The K^trans^ value in patients with post-treatment partial remission and stable disease was significantly decreased compared with patients who continued to progress. This result suggested that K^trans^ value could be an independent index of HCC treatment effects. Jarnagin et al.^15^ found that the variation in the DCE-MRI index during pre-treatment and early post-treatment could predict the therapeutic effect of partial arterial infusion chemotherapy for HCC patients with no surgical resection.

This study in fact shows that the GBCA with different molecular structure delivered different quantitative result in DCE-MRI scan. The Gd-EOB-DTPA with a lipophilic group can enter into the hepatocyte and generated a dual-input three-compartment model: the plasma, intracellular space and extracellular space, which are considered as three separated spaces. Based on the dual-input three-compartment model, more accurate hepatic quantitative analysis should be taken. On the other side, dual-input two-compartment model with non-specific Gd-DPTA should be applied in other area like CNS, prostate, osteoarthritis etc.

In summary, Gd-EOB-DTPA is a liver-specific GBCA that can be taken up by the hepatocytes and excreted by the bile ducts. The K_ep_ and V_e_ using Gd-EOB-DTPA were different from those obtained using a non-specific GBCA due to differences in the excretion pathway between the two types of contrast agents. Therefore, care should be taken when analysing quantitative DCE-MRI data using either type of GBCA.
